# Evaluating large language models in patient education on facial plastic surgery: a standardized protocol

**DOI:** 10.1097/SP9.0000000000000052

**Published:** 2025-06-11

**Authors:** Yousef Tanas, Grace Gasper, Keyvon Rashidi, Sarya Swed

**Affiliations:** a Institute for Reconstructive Surgery, Houston Methodist Hospital, Weill Cornell Medical College, Houston, Texas; b Texas A&M School of Engineering Medicine, Houston, Texas,; c Faculty of Medicine, University of Aleppo, Syria

**Keywords:** large language modelss, patient education, plastic surgery

## Abstract

**Background:**

Large language models (LLMs) are increasingly used in healthcare settings to provide patient education and answer medical inquiries. However, their reliability in delivering accurate, clear, and unbiased information remains uncertain. This study aims to evaluate the quality of responses generated by LLMs to common patient questions regarding facial plastic surgery.

**Methods:**

A total of 60 patient-oriented questions related to facial plastic surgery will be selected from professional bodies, patient support groups, and social media platforms. These questions will be categorized into six main topics: fundamental knowledge, preoperative considerations, surgical procedures, procedural risks and postoperative complications, preparation and recovery, and miscellaneous concerns. Seven LLMs – ChatGPT 4o, Claude, Copilot, DeepSeek, Gemini, Grok, and OpenEvidence – will be tested by inputting each question twice using the “New Chat” feature to assess response consistency. Responses will be evaluated by ten American board-certified plastic surgeons using a structured scoring rubric covering four criteria: accuracy, clarity, completeness, and appropriateness. A standardized scoring system will be employed, and inter-rater reliability will be measured to ensure consistency among evaluators.

**Discussion:**

By systematically assessing the responses of multiple LLMs to patient inquiries on facial plastic surgery, this study will provide insights into their reliability and clinical applicability. Findings may help refine LLM-based tools for patient education and identify areas requiring improvement to ensure safe and effective AI-assisted communication in plastic surgery.

## Introduction

Large language models (LLMs) have emerged as transformative tools in healthcare, offering unprecedented capabilities to process and generate human-like text based on vast datasets. These systems, powered by deep learning algorithms, have rapidly evolved from research prototypes to widely accessible platforms used by healthcare professionals and patients alike^[1,2]^. In the realm of healthcare information dissemination, LLMs present a promising solution to address the growing demand for accessible medical information, particularly in specialized fields such as facial plastic surgery where patient education plays a crucial role in treatment outcomes and satisfaction^[3]^.

The integration of artificial intelligence in healthcare has accelerated considerably, with several studies demonstrating the utility of LLMs in various medical contexts^[4,5]^. Recent advancements such as AlphaFold’s breakthroughs in protein structure prediction and AI-based image models for genomic prediction have demonstrated AI’s transformative role in biomedical research and personalized treatment strategies^[6,7]^. These developments underscore the potential for text-based LLMs to complement existing AI modalities by improving patient education and enhancing clinical communication pathways. Despite their growing adoption, significant concerns persist regarding the reliability, accuracy, and safety of LLM-generated content in sensitive medical domains^[8]^.

In plastic surgery specifically, the landscape of AI applications has expanded rapidly^[9,10]^. Garg *et al*^[11]^ compared the use of two LLMs in aesthetic facial plastic surgery, reporting that while ChatGPT generated more accurate responses and used a higher level of language compared to Google Bard™, both chatbots produced content that exceeded the recommended reading level for patients and lacked accurate source citations.

The advantages of employing LLMs for patient education are substantial. These systems offer 24/7 accessibility, standardized information delivery, and the ability to comprehend natural language queries without requiring patients to navigate complex medical terminology. Furthermore, LLMs can potentially reduce healthcare disparities by providing potentially equal access to medical information regardless of geographic or socioeconomic barriers. Nonetheless, these benefits must be balanced against significant limitations, including the potential to propagate misinformation, reflect biases present in training data, and provide responses that lack contextualization for individual patient circumstances.

As newer LLMs have emerged and existing platforms have undergone significant development over the past year, an updated comparative analysis is essential to guide both healthcare providers and patients. The current literature provides limited insight into the relative performance of different LLM platforms specifically in the context of facial plastic surgery patient education. While studies have explored individual models, a comprehensive comparison across multiple contemporary LLMs using a standardized evaluation framework remains absent from the literature.

Our study aims to address this gap by systematically evaluating seven prominent LLMs – ChatGPT 4o, Claude, Copilot, DeepSeek, Gemini, Grok, and OpenEvidence – in their ability to provide accurate, clear, complete, and reassuring responses to common patient inquiries about facial plastic surgery. By implementing a rigorous assessment protocol with expert evaluation from American board-certified plastic surgeons, this research will contribute to the safe and effective implementation of AI-assisted patient education tools in plastic surgery practice.

## Methods

This prospective study will not seek institutional review board approval because no patient-level data will be involved. The authors declare no affiliation with any LLM developers.

This study will gather 60 questions about facial plastic surgery from sources like professional groups (The American Society of Plastic Surgeons), patient support networks (including websites like Reddit), and social media (including Facebook and Twitter/X) to ensure a variety of patient viewpoints. We will also include the most commonly asked questions from actual patient encounters at our home institution. Three researchers will review these questions, selecting only those directly related to facial procedures. Questions that are repetitive, based on personal opinion, unclear, or not about medical topics will be excluded. The chosen questions will be organized into five categories with ten questions each: pre-surgery inquiries, questions about the surgery itself, risks and complications after surgery, preparation and recovery, and other topics (miscellaneous). Some questions might be reworded for better clarity and grammar. The main goal is to collect questions that patients commonly ask to see how well language models can answer them, such as questions about risks or exercise before and after surgery.

### Inquiries and response generation

This study will test seven LLMs: ChatGPT 4o, Claude, Copilot, DeepSeek, Gemini, Grok, and OpenEvidence. Each of the selected questions will be individually entered into each LLM using a fresh chat session. Following methods used in previous research, each question will be asked twice to see if the LLMs provide consistent answers. All communication with the LLMs will be conducted in English. All questions will be entered the same day in all seven LLMs in May 2025. The chosen date will be stated in the final manuscript as LLMs are continuously updated. The versions of LLMs to be used are as follows: ChatGPT-4o, Claude 3.7 Sonnet, Microsoft M365 Copilot, DeepSeek-v3, Gemini 2.5 Pro, Grok 3, and OpenEvidence (v2.0). Each LLM will be accessed via its publicly available platform.

### Evaluation system

To determine how good the AI-generated answers are to patient questions about facial plastic surgery, a specific evaluation system with four criteria has been developed. These criteria are how correct the information is (accuracy), how easy it is to understand and how suitable it is (clarity and appropriateness), how thorough the answer is (completeness), and how well it addresses the user’s needs and provides a sense of confidence (user engagement and reassurance). Ten plastic surgeons who are certified by a medical board will independently evaluate the responses using a detailed scoring guide. This standardized process is in place to make sure the evaluation is consistent and dependable. To ensure a low risk of bias, all evaluators will be blinded and will not be able to determine which LLM they are assessing.

## Evaluation criteria and scoring system

### Accuracy

Each AI’s answer will be rated on how accurate it is, along with its clarity, appropriateness, completeness, and how well it engages and reassures the user. For Accuracy, we’re checking if the response is factually correct, follows current medical guidelines, and doesn’t contain any medical mistakes or misleading information (Table 1). This will involve looking at whether the answer:

- Matches established medical knowledge and practices.
- Doesn’t include any factual errors or old information.
- Uses medical terms correctly.
- Avoids making guesses or stating things that haven’t been proven.

**Table 1:** Scoring rubric for accuracy

Score	Description
3 (excellent)	Completely accurate, current, and consistent with recognized guidelines. No medical inaccuracies or potentially misleading information.
2 (fair)	Largely correct with only minor errors or slightly outdated language that do not meaningfully affect clinical accuracy.
1 (poor)	Contains serious errors, incorrect medical information, or statements that could lead to patient misunderstanding or harm.

### Clarity and appropriateness

The Clarity and Appropriateness of each AI’s answer will be judged on whether it’s easy to understand, well-organized, avoids using excessively complicated medical language, and gives the right amount of information for someone who isn’t a medical professional (Table 2). We will assess this by looking at the following:

- How well the AI uses simple language that patients can understand while still being medically accurate.
- Whether the explanation is presented in a logical order and is easy to follow.
- If the level of detail is suitable – not too much complicated technical information, but enough to be helpful.

**Table 2:** Scoring rubric for clarity & appropriateness

Score	Description
3 (excellent)	Well-organized and easy to understand, using straightforward language while maintaining accuracy. Avoids unnecessary technical terms or overly complex wording.
2 (fair)	Mostly understandable, but includes some technical jargon, complex wording, or slight confusion in phrasing.
1 (poor)	Difficult to follow due to poor structure, heavy use of medical jargon, or overly simplified responses that lack important details.

### Completeness

The Completeness of the AI’s answer will be checked to see if it fully answers the patient’s question and doesn’t miss any important information (Table 3). We will consider the following points:

- Whether the AI has included all the key aspects of the question in its answer.
- If the explanation is thorough enough and doesn’t leave the patient feeling like they’re missing some information or are still unsure.

**Table 3:** Scoring rubric for completeness

Score	Description
3 (excellent)	Fully answers the question, covering all key points thoroughly and leaving no significant gaps in understanding.
2 (fair)	Provides a generally helpful answer but leaves out some minor details or aspects of the question.
1 (poor)	Fails to include essential information, resulting in a response that lacks necessary clarity or completeness.

### User engagement and reassurance

User Engagement and Reassurance will be judged on how well the AI’s response makes the patient feel more confident and trusting, and if it encourages them to talk more with a healthcare professional (Table 4). During the assessment, the following points will be taken into consideration:

- Whether the AI encourages the patient to feel empowered and suggests they should discuss things further with their doctor.
- If the AI avoids using language that might unnecessarily worry the patient or sounds too overly cautious.

**Table 4:** Scoring rubric for user engagement & reassurance

Score	Description
3 (excellent)	Warm and supportive in tone helps the reader feel reassured and empowered to make informed health decisions.
2 (fair)	Somewhat supportive but lacks strong reassurance or clear encouragement to seek professional help.
1 (poor)	Comes across as impersonal, alarming, or overly clinical – potentially increasing anxiety or confusion.

## Discrepancy resolution mechanism

To ensure objectivity in the evaluations, a structured process will be followed to resolve significant disagreements among the ten plastic surgeon evaluators. Initially, each surgeon will score the responses independently, and the level of agreement across all evaluators will be measured using Fleiss’ Kappa. If the Kappa value indicates strong agreement (>0.75), no further action is needed. For moderate agreement (0.60–0.74), discussion among the evaluators may be initiated. However, in cases of poor agreement (<0.60) or if individual scores for any category differ by more than two points, the evaluators will engage in a discussion to understand their differing rationales. Should disagreements persist after this discussion, an independent expert facial plastic surgeon will act as an eleventh reviewer to provide a final, binding decision on the score.

## Final score calculation

Total score = accuracy + clarity and appropriateness + completeness + user engagement and reassurance (Table 5).

**Table 5:** Scoring rubric for final score calculation

Final score (out of 12)	Interpretation
12	Outstanding: The response is entirely accurate, easy to understand, supportive, and thorough.
10–11	Strong: Generally clear and accurate with only minor flaws.
7–9	Fair: Adequate overall, but requires some improvements.
4–6	Weak: Significant gaps or issues in accuracy, clarity, completeness, or tone.

## LLMs model comparisons and statistical analysis

Our main way of scoring the AI responses will give the same importance to all four areas: accuracy, clarity and appropriateness, completeness, and how well it engages and reassures the user. However, we understand that not all of these might be equally important in a real-world medical setting. Specifically, accuracy is likely the most critical factor when it comes to educating patients and ensuring their safety. Because of this, we also plan to do a second analysis where we give different weights to the scores. In this analysis, we will put more emphasis on accuracy and completeness because these directly relate to how correct and thorough the medical information is. We will do this by multiplying the scores for accuracy and completeness by 1.5, while the scores for clarity and user engagement will stay the same (multiplied by 1.0). This will help us see how giving different levels of importance to these areas might change how the AI models rank and give us a more practical understanding of how well they perform in providing important educational information, especially in the context of facial plastics surgery counseling.

To compare the performance of the different AI models (ChatGPT 4o, Claude, Copilot, DeepSeek, Gemini, Grok, and OpenEvidence), the study will first analyze the mean total scores achieved by each model. Statistical significance of any observed differences in these mean scores will be assessed using a One-way ANOVA test, or a Kruskal–Wallis test if the assumptions of ANOVA are not met, with a Bonferroni correction applied to account for the multiple comparisons being made. To ensure sufficient statistical power, a total of 100 responses from each model will be evaluated by each of the ten board-certified plastic surgeons. Finally, the level of agreement among these ten evaluators will be quantified using Fleiss’ Kappa.

## Discussion

Recent studies show that LLMs can often provide patient-facing medical answers of surprisingly high quality, though performance varies by model and context. In fact, AI-generated responses have at times matched or exceeded physician answers in rigor and tone. For example, a cross-sectional trial found that ChatGPT’s answers to patient questions on an online forum were rated significantly higher in quality and empathy than responses from licensed doctors^[12]^. Likewise, an oncology-focused study reported that the best-performing chatbot (Claude AI) produced answers with greater overall quality, empathy, and readability than those given by cancer specialists^[13]^.

In plastic surgery domains, early evaluations are cautiously optimistic: ChatGPT’s explanations about common breast procedures were deemed “sufficient” in quality^[14]^, and it appropriately emphasized urgent care for serious rhinoplasty complications (e.g., infection) while advising watchful monitoring for milder issues^[15]^. Nonetheless, not all outputs are correct or complete – one assessment of emergency-care queries showed that LLM responses were only about 50% accurate and comprehensive, and up to a third of answers contained some unsafe or inappropriate advice^[16]^. This variability shows that while LLMs can deliver accurate medical information, they also risk important omissions or errors in certain scenarios.

A key challenge for AI in patient education is ensuring responses are understandable and suitably tailored. LLM-generated answers are typically fluent and well-structured – the breast surgery study noted ChatGPT provided highly organized content despite omitting some specifics^[14]^. Moreover, these models often adopt a friendly, empathetic tone. Physicians reviewing answers have praised ChatGPT for its compassionate wording, rating its bedside manner far above that of real clinicians in some cases^[12]^. Yet readability remains a concern. Multiple evaluations reveal that ChatGPT’s default explanations overshoot recommended reading levels for the general public^[17]^. For instance, answers from ChatGPT-3.5 about ACL injuries averaged around a 12th-grade reading level^[17]^, and a comparison in foot and ankle surgery found AI-generated pamphlets required ~11th-grade comprehension versus ~1st-grade for professionally written leaflets^[18]^. Not all LLMs are equally difficult: Google’s Bard [now Gemini] tends to produce simpler text. In a CPR education study, Bard’s [Gemini’s] responses were the easiest to read (roughly 8th–9th grade), while ChatGPT’s were significantly more complex^[19]^. Similarly, for cataract surgery questions Bard delivered the most readable answers, whereas ChatGPT’s replies, though more detailed, were harder to digest.

Despite promising performance, current LLMs suffer from important limitations. Many models do not consistently cite medical sources and may generate hallucinated references. They lack real-time clinical updates and struggle with personalized medical recommendations. Additionally, the proprietary nature of training data and algorithms introduces opacity that may undermine clinical trust.

One stark difference among LLM-based tools is how they handle sources and citations, which in turn affects user trust. Standard ChatGPT and Bard [Gemini] outputs usually do not cite references, and their responses lack transparent authorship or date information nearly 100% of the time^[19]^. If asked to provide sources, these models often struggle; studies have documented frequent “hallucinated” citations – references that look plausible but are non-existent. In a head-to-head evaluation of reference generation, Bard was especially prone to fabrication, with over 90% of its citations being false. Even ChatGPT (GPT-3.5/4) produced a substantial number of bogus or irrelevant sources in that analysis^[20]^.

Given these potential limitations, researchers overwhelmingly caution that current LLMs should supplement rather than replace professional medical advice^[14]^. Many emphasize that while AI can increase accessibility to health information, it lacks accountability and up-to-date domain nuance^[21]^. In the context of plastic surgery, experts concur that chatbots might help educate patients and improve baseline knowledge, but their role must remain ancillary to a surgeon’s guidance^[14]^. Thus, the aim of our study, following this protocol, is to help the most suitable LLM for patient education in facial plastic surgery.

It is essential to emphasize that LLM outputs should not substitute for professional medical advice. Dissemination of AI-generated content must include clear disclaimers that reinforce the role of licensed healthcare providers in patient care.

## Data Availability

Datasets generated during and/or analyzed during the current study are publicly available.
